# Polymerization-Induced Phase Separation Formation of Structured Hydrogel Particles via Microfluidics for Scar Therapeutics

**DOI:** 10.1038/s41598-018-20516-9

**Published:** 2018-02-02

**Authors:** S. Guo, G. Kang, D. T. Phan, M. N. Hsu, Y. C. Por, C. H. Chen

**Affiliations:** 1 Department of Biomedical Engineering, National University of Singapore, 4 Engineering Drive 3, 117583 Singapore, Singapore; 2 Department of Plastic, Reconstructive & Aesthetic Surgery, KK Women’s and Children’s Hospital, 100 Bukit Timah Rd, 229899 Singapore, Singapore; 3 Biomedical Institute for Global Health Research and Technology (BIGHEART), National University of Singapore, 14 Medical Drive, 117599 Singapore, Singapore; 4 Singapore Institute of Neurotechnology (SINAPSE), National University of Singapore, 28 Medical Drive, 117456 Singapore, Singapore

## Abstract

Excessive scar formation can form disabling contractures that result in a debilitating psychological outcome. Sustainable hydrophobic corticosteroid release *in vivo* is essential to regulate the wound healing process. Functional hydrogel particles are widely applied for sustainable release. However, due to the limited aqueous solubility of hydrophobic compounds, most of the corticosteroid is released from the hydrogels within seconds, causing undesirable scar formation and recurrence. In this study, a novel polymerization-induced phase separation is investigated to form well-defined polyethylene glycol diacrylate (PEGDA) core/alginate shell structured hydrogel particles using microfluidics without toxic organic solvents. Based on their wettability preference, hydrophobic corticosteroid-loaded poly(lactic-co-glycolic acid) (PLGA) nanoparticles are compartmentalized in the PEGDA core during polymerization to control the corticosteroid release. The distribution of the PLGA nanoparticles is precisely regulated by the phase separation boundary and characterized using a fluorescent dye. The thickness of the shell and partition coefficients are determined using the UV intensity and irradiation period. Upon encapsulation of the PLGA nanoparticles within the poly(PEGDA) core, a long-term corticosteroid treatment is developed and effective scar therapeutic outcomes are evaluated using both *in vitro* and *in vivo* models.

## Introduction

Scars are areas of fibrous tissue that replace normal skin during the wound healing process. With the exception of minor lesions, wounds result in some degree of scarring on skin. Excessive scar formation, such as keloids, is due to an abnormal response to injury, and the formation of disabling contractures and poor cosmesis cause debilitating psychological outcomes^1^. Hydrophobic drug treatments, such as corticosteroids and triamcinolone, are essential to reduce the glycosaminoglycan synthesis, inflammation and fibroblast proliferation^2^. Current protocols include painful monthly injections to administer a bolus dose of the medication into the scar^3^. Unfortunately, the recurrence rates are as high as 50% upon cessation of these injections^4^. When triamcinolone acetonide (TA) is used, a high initial dose or frequent TA injections must be administered to the patients to maintain a high TA concentration in the target tissue for prolonged periods, causing high cost for the treatment (~200 USD per drug loading process) and undesirable side effects^5^.

To overcome the challenges of hydrophobic drug delivery, a wide range of different nanocarriers have been developed^6–10^. For example, poly(lactide-co-glycolide) (PLGA) nanoparticles were developed for hydrophobic reagent delivery with a tailored biodegradation rate (depending on the molecular weight and copolymer ratio), but a burst release of the cargo without controls was observed^11–13^. To prevent a burst release, PLGA was co-polymerized with polycaprolactone (PCL) and polyethylene glycol (PEG) to form (PLA-PCL-PEG-PCL-PLA) and offer a promising controllable release profile for a hydrophobic drug^14^. However, the biodegradability of the system was affected by the conjugation of the PCL segment because of its hydrophobic, crystalline nature.

A hydrogel is highly biocompatible and biodegradable and contains a three-dimensional porous network that can encapsulate a large reagent amount for long-term delivery^15–19^. However, due to the hydrophilic nature of the polymeric network in a hydrogel, challenges remain in regulating the release profiles of hydrophobic reagents^20^. To address this issue, a variety of strategies have been investigated. For example, a mono-acrylated PEG hydrogel with a carboxylic acid group was developed and conjugated with the primary hydroxyl group on dexamethasone for the controlled release of hydrophobic reagents^21^. However, this system can only encapsulate a drug with a primary hydroxyl group base via an esterification reaction. A homogeneous hydrogel matrix with PLGA nanoparticles loaded with hydrophobic drugs was developed as a universal platform for hydrophobic drug delivery, but a burst release profile was observed for the loaded hydrophobic drug from the hydrogel matrix^22–24^. Therefore, optimized platforms for sustainable hydrophobic drug delivery with a controllable release profile are not available for long-term scar therapeutics.

In this study, core-shell structured hydrogel particles fabricated using microfluidics were developed as a universal sustainable hydrophobic reagent/drug delivery system for effective low cost scar therapeutics. To fabricate core-shell structured hydrogel particles, droplet based microfluidic process was developed before^25–30^. Double emulsions produced via microfluidics were used as the templates to form core-shell hydrogel particles^30–32^. However, this procedure forming double emulsions was complicate. The wettability of the device needs to be patterned containing hydrophilic part (forming hydrophobic droplets) and hydrophilic part (forming hydrophilic droplets) to make water-in-oil-in-water (or oil-in-water-in-oil) double emulsions^30^. After that, the synchronization should be performed for encapsulation of monomer droplets, forming structured hydrogel particles after polymerization^30,31^. Above to complicate procedure of fabrication, none of current technologies can be used to spontaneously compartmentalize hydrophobic reagents in one compartment for long term releasing.

Here, a simple polymerization-induced phase separation (PIPS) of an aqueous two-phase system (ATPS) was introduced to form biodegradable core-shell structured hydrogel particles using one step droplet formation via microfluidics. The monodispersed droplets were produced by flowing monomers and oil in a co-flow device without complicated microfluidic operations. After that, homogeneous droplets containing PEGDA, alginate, EDTA-Ca and PLGA nanoparticles phase separated to form core-shell structured hydrogel particles after the polymerization of PEGDA^33^. Simultaneously, corticosteroid-loaded PLGA nanoparticles spontaneously moved to the core of the hydrogel particle because of the ATPS extraction^34^. Accordingly, after gelation of the alginate shells upon the injection of acetic acid^35^, the structured pre-gel droplets converted into core (PEGDA/PLGA)-shell (alginate) structured hydrogel particles with concentrated PLGA nanoparticles in the PEGDA core. The well-defined core-shell structure regulated the burst release of the hydrophobic reagents. The partition ratio of the PLGA nanoparticles indicated 98% encapsulation of the corticosteroids in the PEGDA cores for long-term release. The burst release in the core-shell hydrogels was ~40%, which was in contrast to the release without the core-shell hydrogel encapsulation (~60%). The corticosteroids (80~85%) were released in a well-controlled manner over 30 days. Rabbit ears were used as the *in vivo* model. Based on the histologic analysis, the scar elevation index (SEI), which is an accurate instrument used to evaluate hypertrophic scar formation, showed a significant reduction (to ~1.28) with the injection of the core-shell hydrogel particles.

## Results and Discussion

### Polymerization Induced Phase Separation in the Droplets

**Figure 1:**
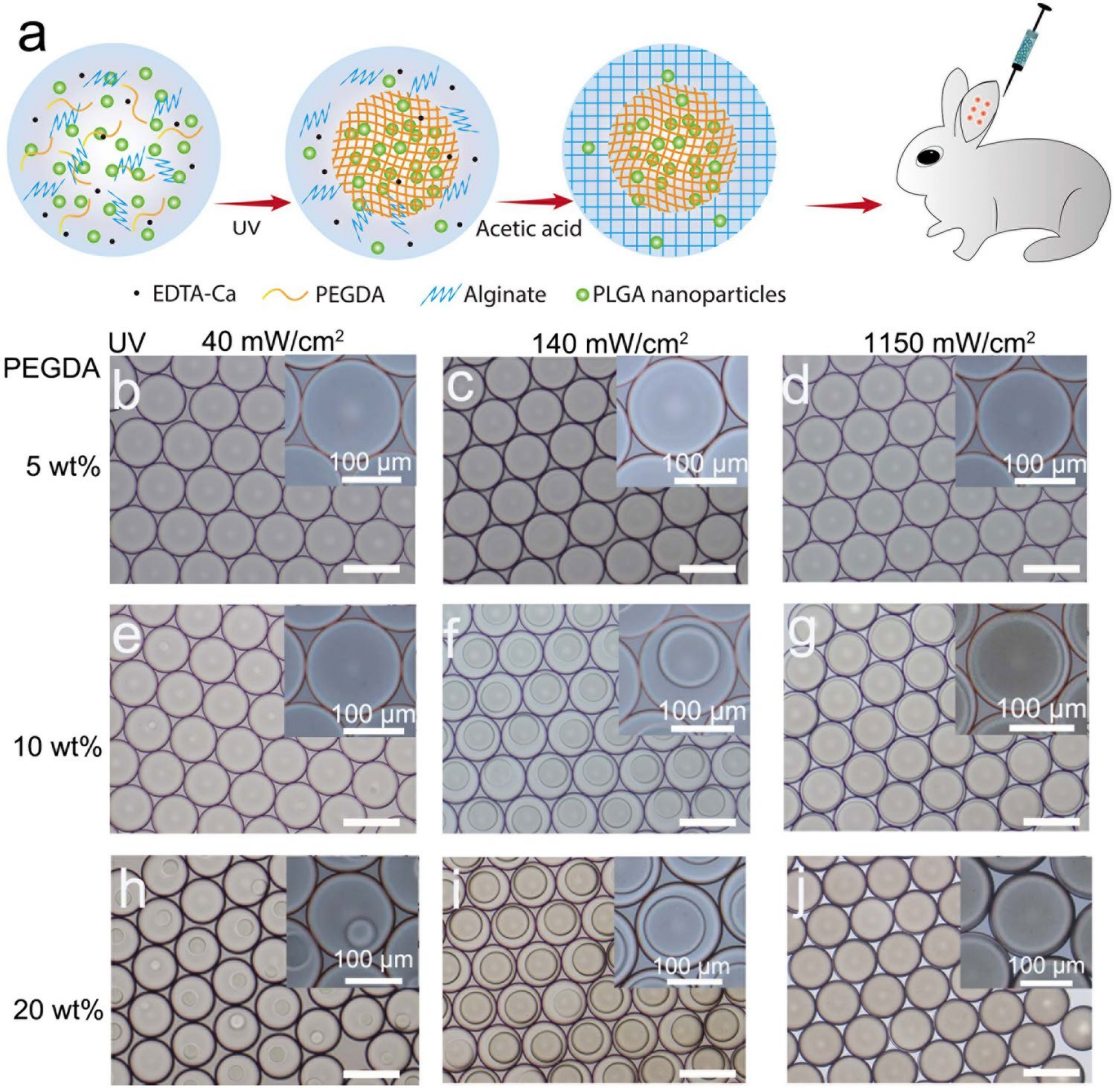
(**a**) Scheme of the core-shell hydrogel particle fabrication process. PIPS to form core-shell structured hydrogel particles via microfluidics for long term hydrophobic reagent delivery. (**b**–**j**) Changing the UV intensity resulted in the different core diameters. When the UV intensity increased, the diameter of the cores increased. In this experiment, the flow rates of the oil and aqueous phases were at 30 and 5 μl/min, respectively, in all the experiments. The average diameter of the droplets was 158 μm. The scale bar is 200 μm.

The synthesis route of the structured hydrogel particles was schematically illustrated in Fig. 1a. To characterize the phase separation during the polymerization in the droplets, the PIPS of the PEGDA aqueous droplets, which contained PEGDA, the photo initiator, alginate, EDTA-Ca and H_2_O, was investigated. The aqueous droplets were polymerized via UV irradiation in the microchannel. Monodispersed pre-gel droplets containing PEGDA (Mn = 575), alginate, PLGA nanoparticles (loaded with drug), the photo-initiator and EDTA-Ca were irradiated by UV light for polymerization and subsequent formation of the hydrogel particles. Due to PIPS, PEGDA polymerized and formed a round hydrogel core in the aqueous droplet. Based on the extraction of the aqueous system, the PLGA nanoparticles spontaneously distributed into the poly(PEGDA) core during the phase separation process. Then, for future gel aqueous droplets, HFE 7500 with 0.5 vol% acetic acid was used to trigger the subsequent crosslinking of alginate to form PEGDA core-alginate shell hydrogel particles for sustainable hydrophobic drug treatment *in vivo*.

The as-formed structured droplets were collected, as shown in Fig. 1b–j. The error bars are used to indicate the standard deviation. When the concentration of the PEGDA increased from 5 wt% to 10 and 20 wt%, dense poly(PEGDA) cores with well-defined structures formed. The size of the poly(PEGDA) core can be controlled by tuning the UV light intensity. When the PEGDA concentration was 5 wt%, the diameter of the poly(PEGDA) core increased from 21 μm to 131 μm as the UV light intensity increased from 40 mW/cm^2^ to 1150 mW/cm^2^. This can be attributed to PEGDA monomer conversion increased as UV intensity increased (supporting information, section 1, Table S1). It is worth to note that in this polymerization process, a radical procedure of the propagating free radical chain end to undergo a variety of different termination reactions was applied to form poly-disperse polymers with limited control over macromolecular weight and architecture^36^. When higher UV intensity was applied, more monomers were polymerized, forming larger core (increasing the diameter of the core), which was consist with the previous work (supporting information, section 2, Figure S4)^33^.

### The Partitioning of the PLGA Nanoparticles during the PEGDA Polymerization

**Figure 2:**
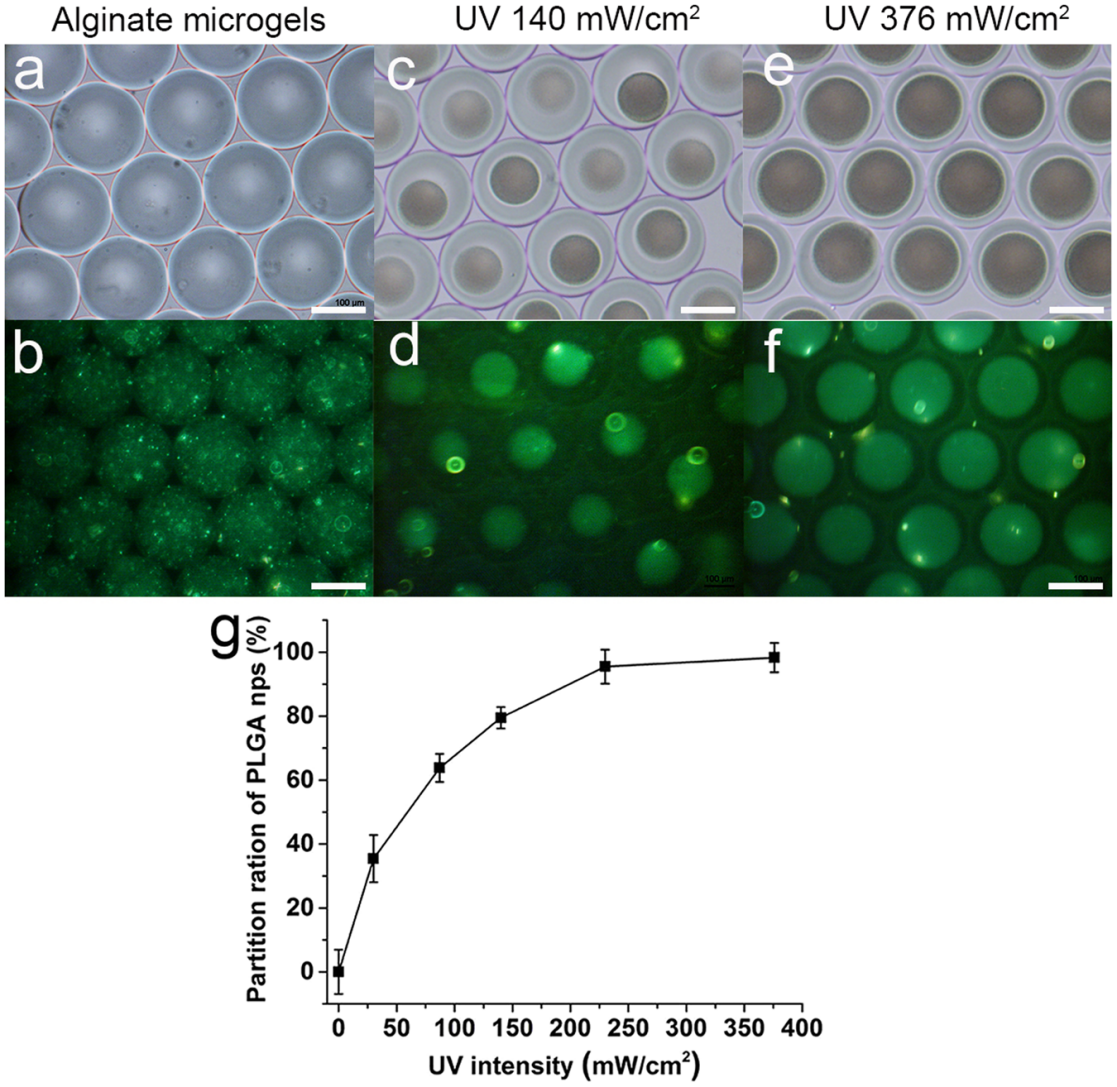
Microscope bright field and fluorescence images of the alginate hydrogel particles (**a**,**b**) and core-shell hydrogel particles prepared with different UV intensities (**c**–**f**) were recorded. The PLGA nanoparticles were loaded with coumarin 6 and showed a green color in the fluorescence images. The scar bar is 100 μm. (**g**) The partition ratio of the PLGA nanoparticles in the core-shell hydrogel particles. The calculation details are provided in the supporting information (section 3). The error bars presented are the standard deviations.

Before the PEGDA polymerization, aqueous droplets with a homogeneous mixture of coumarin 6-loaded PLGA nanoparticles, the PEGDA monomer (10 wt%), alginate (1.0 wt%), EDTA-Ca (0.05 wt%) and the photoinitiator (0.5 wt%) were observed via a bright field image, and the image showed transparent droplets (Fig. 2a) and green fluorescence (Fig. 2b). As shown in Fig. 2c, PEGDA was polymerized in 3 seconds by UV irradiation with an intensity of 140 mW/cm^2^, and this triggered the phase separation between the poly(PEGDA) and the aqueous solution in the droplets in 3 seconds to form structured droplets with poly(PEGDA) cores (diameter 86 μm). Using coumarin 6, the partitioning of the PLGA nanoparticles was observed via fluorescence emission, and the droplets are shown with bright cores and dark shells in Fig. 2d. The PLGA nanoparticles were spontaneously and selectively trapped within the PEGDA cores due to their similar wettability. Increasing the UV irradiation intensity to 376 mW/cm^2^ increased the core size to 131 μm (Fig. 2e). Accordingly, the distribution of the PLGA nanoparticles in the droplets changed, as shown in Fig. 2f.

The partition ratio of the PLGA nanoparticles in the hydrogel particles was evaluated by measuring the fluorescence intensity of the alginate shell (Fig. 2b,d and f). The partitioning of the PLGA nanoparticles into the poly(PEGDA) core depended on the UV irradiation intensity (for the same exposures time, ~3 seconds). For example, with a UV irradiation intensity of 140 mW/cm^2^, 79.5% of the PLGA nanoparticles were encapsulated in the poly(PEGDA) core of the structured hydrogel particles. When the UV irradiation intensity increased to 376 mW/cm^2^, the partitioning of the PLGA nanoparticles into the poly(PEGDA) core in a hydrogel particle increased to 98.5%.

### Sustainable Hydrophobic Drug Release Profile

**Figure 3:**
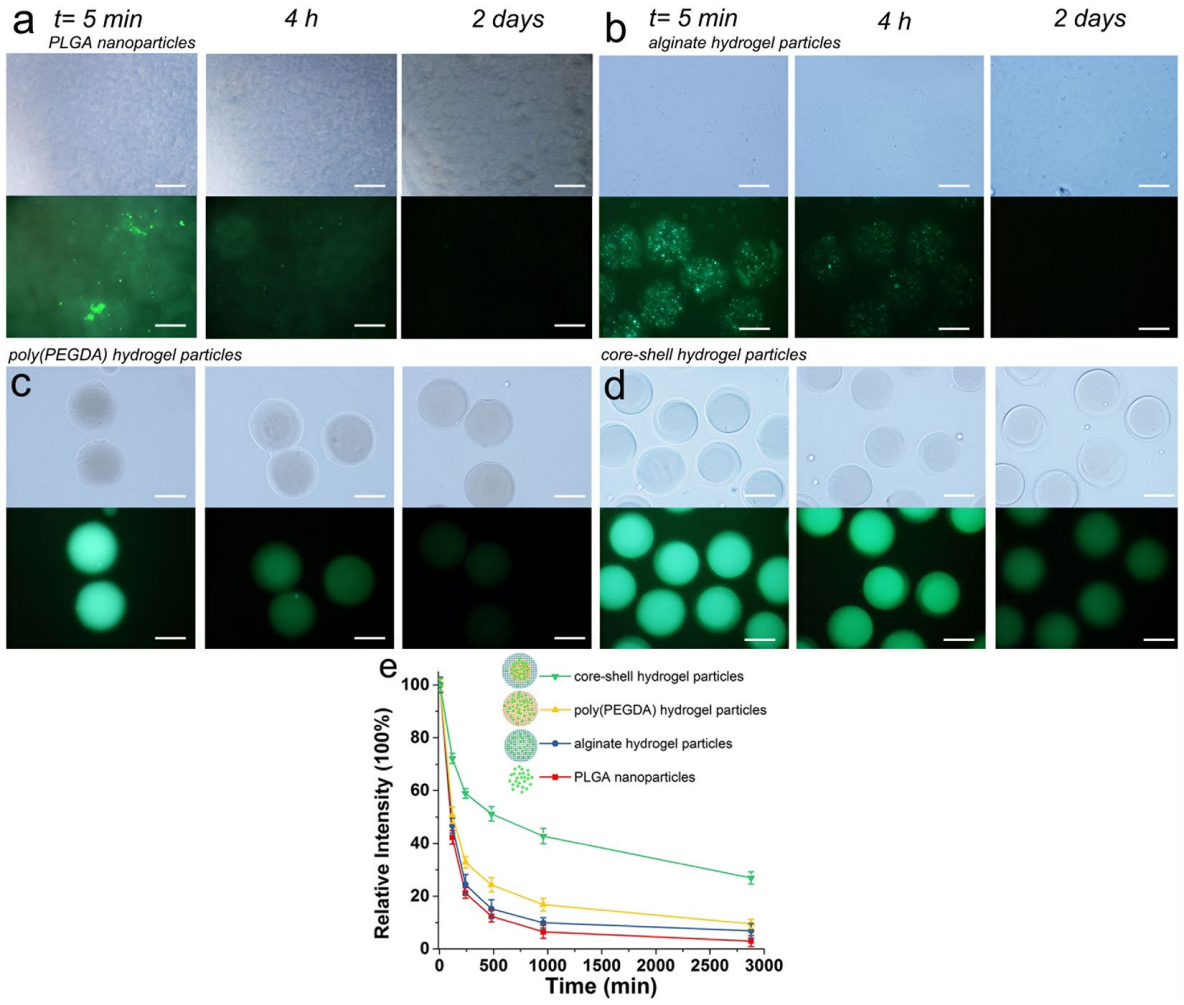
The bright field and fluorescence images showing the release process of coumarin 6 from the PLGA nanoparticles (**a**), alginate hydrogel particles (**b**), poly(PEGDA) hydrogel particles (**c**) and core-shell hydrogel particles (**d**) in PBS (pH 7.4, 37 °C). (**e**) The release profile of coumarin 6 obtained from the fluorescence images. The inserted scar bar is 100 μm. The error bars presented are the standard deviations.

Coumarin 6 is a hydrophobic dye that is used as a model hydrophobic reagent to investigate the release of hydrophobic reagents from prepared structured hydrogel particles. The release profiles of the PLGA nanoparticles, the alginate hydrogel particles (the shell) and the poly(PEGDA) hydrogel particle (the core) in a PBS solution were characterized for comparison. Due to the wettability difference between coumarin 6 and aqueous solutions, coumarin 6 was released from the PLGA nanoparticles within 2 days, as shown in Fig. 3a. The dye release was slightly regulated by encapsulating the hydrophobic dye-loaded PLGA nanoparticles in the alginate hydrogel particles, but the hydrogel particle fluorescence intensity dramatically decreased to 24.4% in 4 h (Fig. 3b and e), indicating the burst release of coumarin 6. After 2 days, the fluorescence signal in the alginate hydrogel particles was very weak. The same burst release of the hydrophobic reagents was observed in the PEGDA hydrogel particles with the PLGA nanoparticle encapsulation (Fig. 3c). In comparison, although a similar burst release was observed in the first 4 h in the core/shell structured hydrogel particles, the particles retained a high fluorescence intensity, which showed a sustainable hydrophobic reagent release (Fig. 3d). The fluorescence intensity decreases in the four systems were quantitatively analyzed (Fig. 3e) to determine the hydrophobic reagent release profile of the core-shell hydrogel particles.

**Figure 4:**
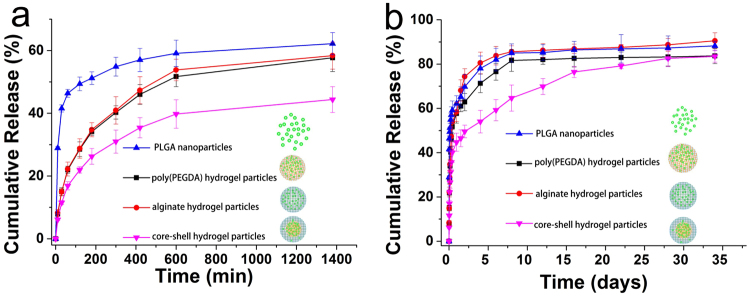
(**a**) Release profiles for TA from the PLGA nanoparticles, PEGDA hydrogel particles, alginate hydrogel particles and core-shell hydrogel particles for the first 1440 min (1 day) and (**b**) the release profile for 1 month in PBS (pH 7.4, 37 °C). The error bars presented are the standard deviations.

To investigate the sustainable hydrophobic drug release profile, TA was loaded into the four systems (PLGA nanoparticles, alginate hydrogel particles, poly(PEGDA) hydrogel particles and core-shell hydrogel particles). The loaded TA concentration was 2.5 mg/ml, and the release profiles were measured by HPLC with a UV/VIS detector in a PBS solution at pH 7.4 and 37 °C. The release kinetics of TA in the four systems were investigated on the first day and are shown in Fig. 4a. The burst release of TA from the PLGA nanoparticles, poly(PEGDA) hydrogel particles and alginate hydrogel particles was observed in the first 600 min. Because of the wettability difference, the PLGA nanoparticles released ~60% of the hydrophobic drug (TA). After the first day, the release from the PLGA nanoparticles was ~63%. Once the PLGA nanoparticles were encapsulated by the poly(PEGDA) hydrogel particles via microfluidics, the burst release in the first 600 min was regulated to ~50%. After the first day, the release from the poly(PEGDA) was ~55%. A similar reduction in the burst release was observed when the PLGA nanoparticles were encapsulated by the alginate hydrogel particles. Compared with the PLGA nanoparticles, the poly(PEGDA) hydrogel particles and the alginate hydrogel particles, the burst release of TA from the core-shell structured hydrogel particles was limited to 40% in the first day because the PLGA nanoparticles were encapsulated in the poly(PEGDA) core and the alginate shell served as a barrier to inhibit the diffusion between the poly(PEGDA) core and the PBS solution. The long-term TA release profile for approximately one month (34 days) is shown in Fig. 4b. In the PLGA nanoparticles, the poly(PEGDA) hydrogel particles and the alginate hydrogel particles, 80% of the TA was released within 8 days. In contrast, the release rate for the core-shell hydrogel particles was nearly linear and well-controlled between the first day and 28 days (~27 days). After 30 days, the total release of TA (~85%) was similar for all the systems (PLGA nanoparticles, poly(PEGDA) hydrogel particles, alginate hydrogel particles and core-shell hydrogel particles).

### Long-term *in vivo* scar therapeutics

**Figure 5:**
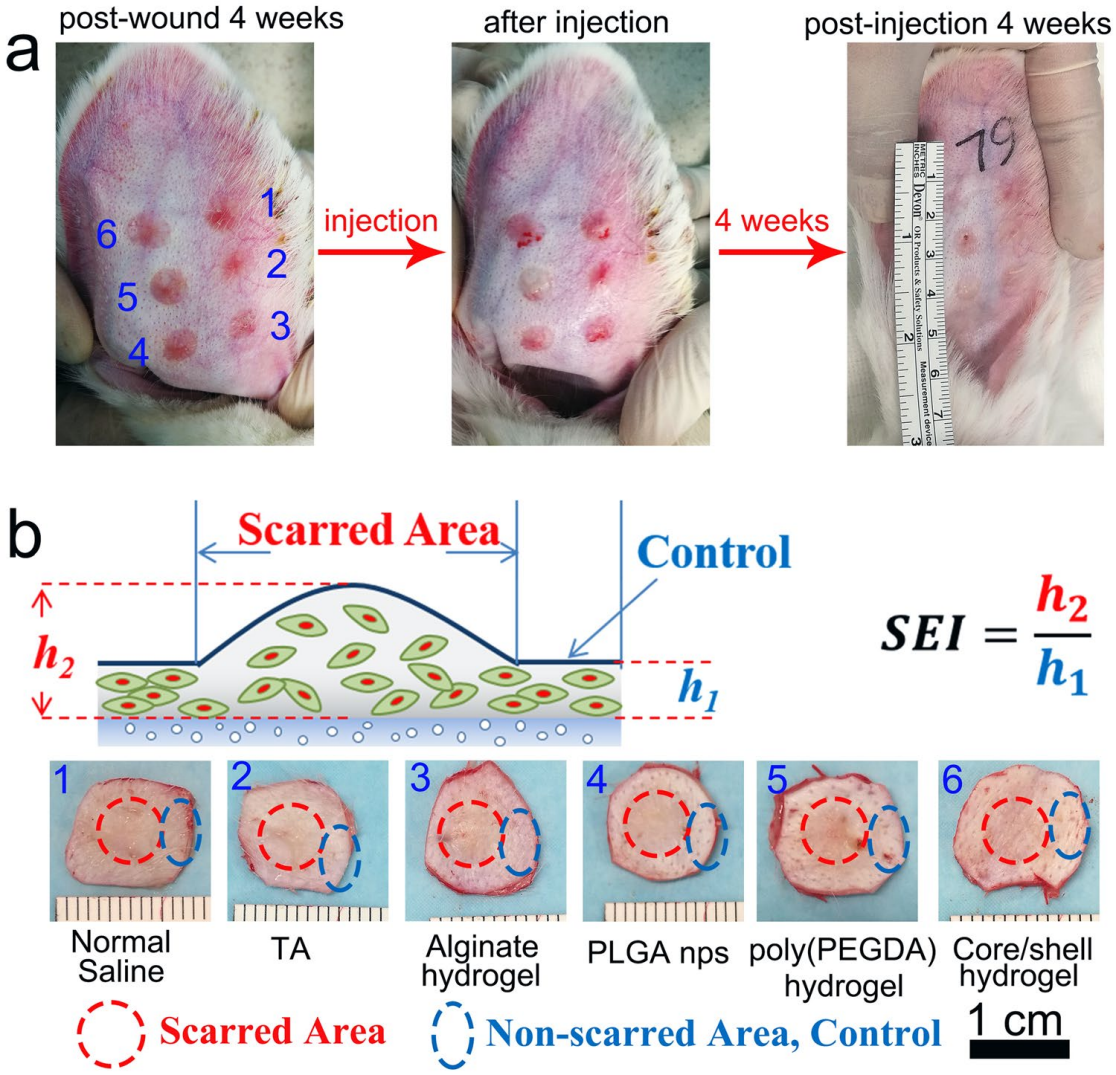
(**a**) Six hypertrophic scars on the ventral side of a rabbit ear 4 weeks’ post-wound. After that, the different formulas were injected. The scars were harvested 4 weeks’ post-injection. (**b**) Definition of SEI was presented. Scarred areas were marked by red circles. Normal tissue areas (non-scarred areas) were marked by blue circles. *h*_1_ was the maximum thickness of the dermis in non-scarred area (control). *h*_2_ was the maximum thickness of the dermis in scarred area. The bar inserted was 1 mm.

To evaluate the potential for sustainable TA therapeutics using core-shell structured hydrogel particles to prevent hypertrophic scar formation, we injected suspensions of these particles into the hypertrophic scars on the rabbits’ ears for long-term observation (Fig. 5a). The well-defined scar areas on the rabbit ears were prepared using a puncher (diameter 6 mm), as described in the Methods Section. The therapeutic efficiencies of five different systems were tested using the rabbit ears as the *in vivo* models: (1) core-shell hydrogel particles containing TA-loaded PLGA nanoparticles, (2) poly(PEGDA) hydrogel particles containing TA-loaded PLGA nanoparticles, (3) alginate hydrogel particles containing TA-loaded PLGA nanoparticles, (4) a suspension of TA-loaded PLGA nanoparticles, (5) a TA suspension. The TA concentration was 2.5 mg/ml in all the samples, and the samples were suspended in saline. An additional group of ears was injected with saline alone as the control experiment. The wound healing process and scar formation on the rabbit ears were recorded every 7 days for 4 weeks (28 days). To observe the scar formation details, these rabbit ears were harvested and stained by hematoxylin and eosin (H&E) to mark the skin tissues for observation under a microscope after four weeks of treatment. Scar Elevation Index (SEI) was defined by the ratio of the tissue height in the wound area and the tissue height of the normal tissue area around the scar (*h*_2_*/h*_1_) to quantify the therapeutic efficiency. The definitions of *h*_1_ and *h*_2_ were the maximum thickness of the dermis in non-scarred area (control) and scarred area, respectively. To evaluate SEI value of the scars, in Fig. 5b, both scarred area and normal tissue (non-scarred, control) area were identified in each image. The central area marked by a red circle was the scarred area. The surrounding area marked by a blue circle was the non-scarred area as the control. Foreign body reactions of alginate and poly(PEGDA) were examined *in vivo* (section 5, supporting information, Figure S8) to suggest the foreign body reactions were not significantly affect scar treatment. (Figure S9, supporting information).

**Figure 6:**
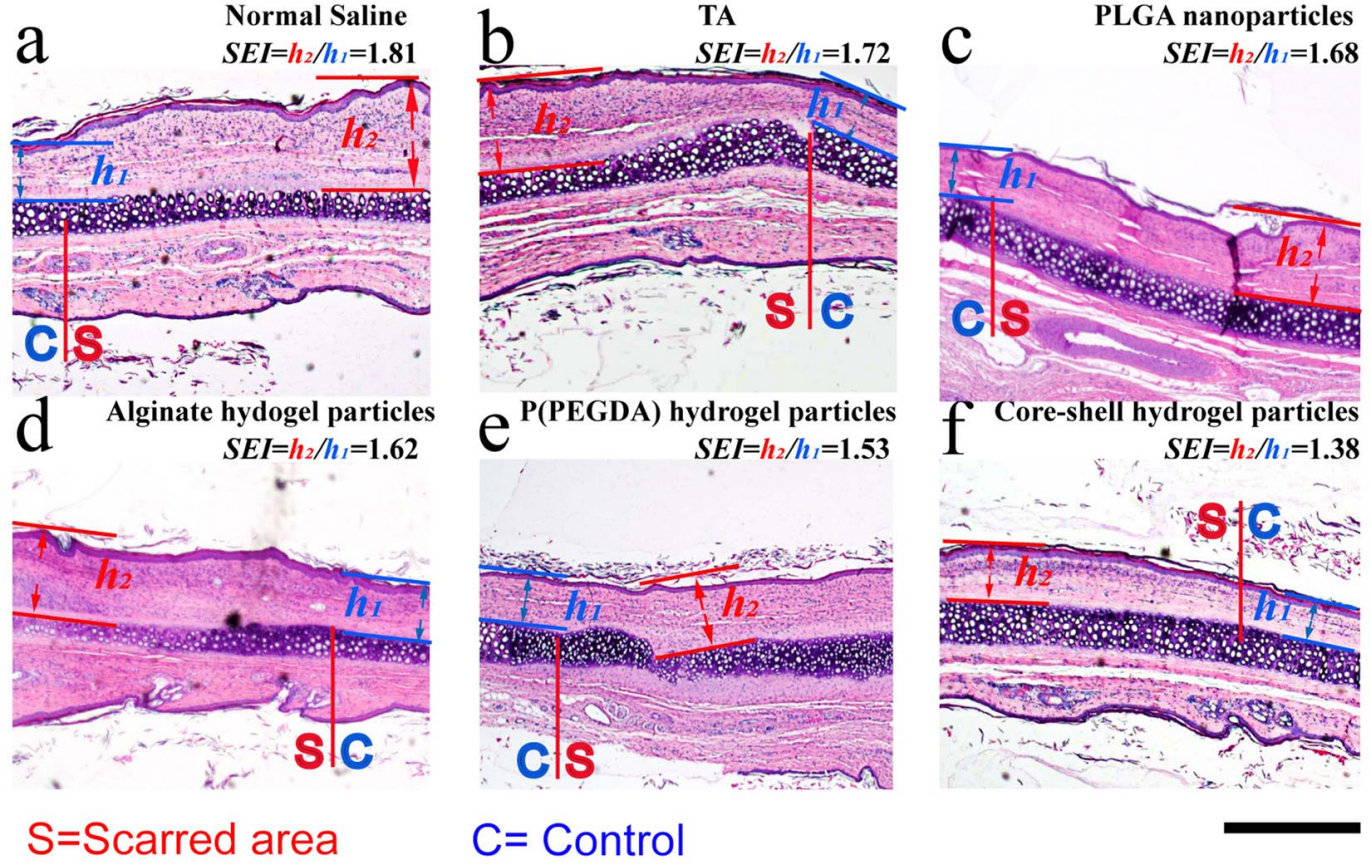
H&E histological analysis of the scars harvested 4 weeks’ post-injection. (**a**) Normal saline-treated control scar; (**b**) TA suspension-treated scar; (**c**) PLGA nanoparticle-treated scar; (**d**) alginate hydrogel particle-treated scar; (**e**) poly(PEGDA) hydrogel particle-treated scar. (**f**) Core-shell hydrogel particle-treated scar. *h*_1_ is the dermal thickness in the non-scarred area (control). *h*_2_ is the dermal thickness in the scarred area. The insert bar represents 1 mm.

**Figure 7:**
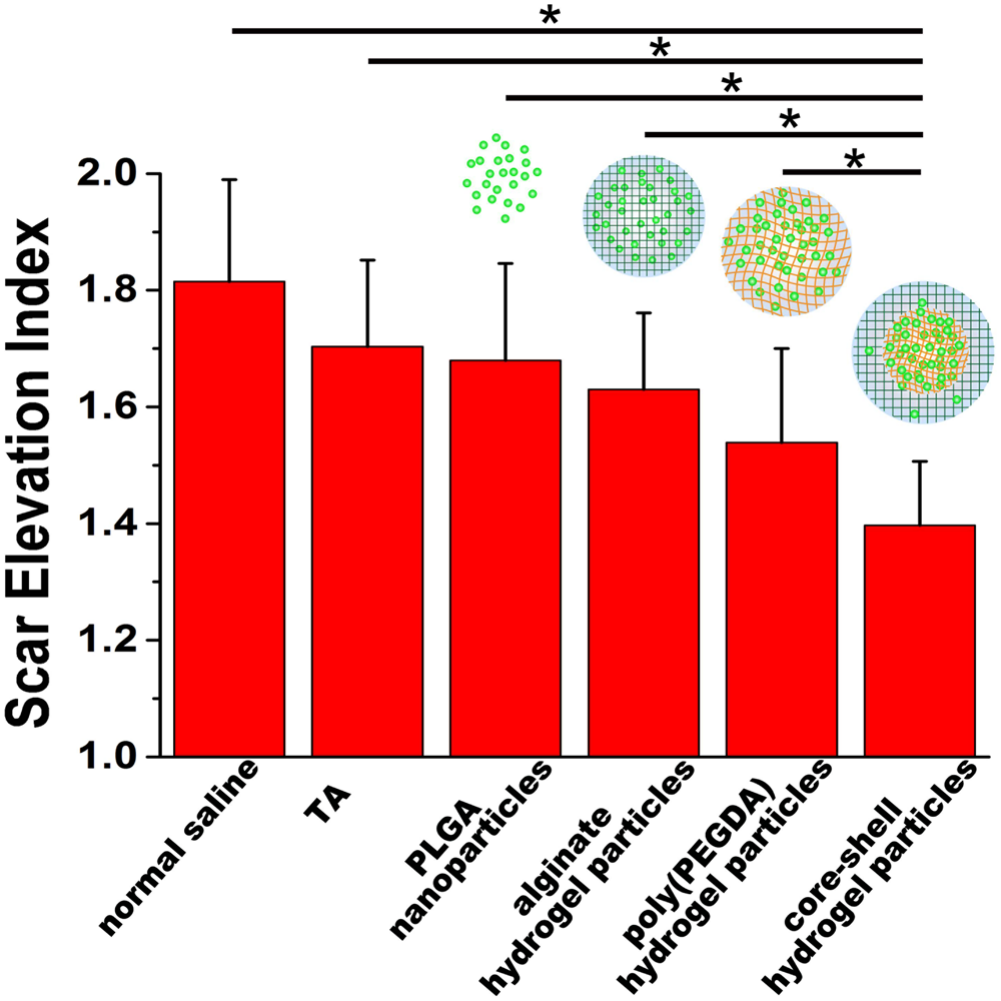
SEI data for the wounds treated with normal saline, a TA suspension, the PLGA nanoparticles, the alginate hydrogel particles, the poly(PEGDA) hydrogel particles and the core/shell hydrogel particles. *p < 0.05, n = 12. Data presented mean ± standard deviations.

The H&E dye reveals the histological features of the scar in the harvested rabbit ears under a microscope^37^, as shown in Fig. 6. In the normal tissue (non-scarred) area, dermal thickness *h*_1_ was measured as the control. In the scarred area, abundant, horizontally arranged collagen fibers, occasionally in a whorled or circular configuration, with increased vascularity and chronic inflammation were observed to indicate the formation of a hypertrophic scar with dermal thickness *h*_2_ and rough surface^2,3^. The features of scars were mitigated in the wounds treated with the hydrogel particles, as evidenced by the decrease in the dermal thickening and fewer irregular collagen fibers. Scars treated with the TA suspension and the PLGA nanoparticles showed a decrease in the SEI after 4 weeks (1.71 ± 0.14 and 1.68 ± 0.13, respectively), which indicated TA inhibited the hypertrophic scar formation (Fig. 7 and Table S3 in Supplementary information). Using the alginate hydrogel particles and poly(PEGDA) hydrogel particles as carriers to regulate the TA release profile resulted in a reduction in the height of the hypertrophic area (i.e., the area above the normal skin height), and decreases of 22% and 28%, respectively, compared to the control experiments (1.81 ± 0.18) were observed. The long-term sustained release of TA from the core-shell structured hydrogel particles resulted in a 52% reduction in the scar area (1.39 ± 0.11, estimated by SEI) compared to the control experiments.

## Conclusion

In this study, PIPS was used to fabricate novel poly(PEGDA) core/alginate shell structured hydrogel particles using microfluidics. During the PEGDA polymerization, the poly(PEGDA) cores encapsulated the hydrophobic corticosteroid loaded PLGA nanoparticles and spontaneously separated from the alginate solution to form core-shell structured droplets. Mixing the structured droplets with an oil phase (containing acetic acid) caused the alginate to crosslink, and the structured hydrogel particles were obtained. The PLGA nanoparticles inside the core inhibited the burst release, and a sustainable hydrophobic corticosteroid release over 4 weeks was achieved. A rabbit ear model was used for the *in vivo* tests, and effective therapeutic outcomes to suppress scar formation were exhibited. The H&E histological analysis of the harvested scars showed that the wound healing process was well-controlled by implanting the core-shell hydrogel particles with hydrophobic corticosteroid-loaded PLGA nanoparticles. An SEI analysis was performed. The wounds treated with the core-shell hydrogel particles had the lowest SEI values, which confirmed the core-shell hydrogel particles inhibited scar formation. The core-shell structured hydrogel particles have the ability to compartmentalize PEGDA with corticosteroids and other hydrophobic drugs, and the particles are a generic and useful system for the regulation of hydrophobic drug delivery and long-term therapeutics.

## Methods

### Synthesis of Drug-loaded PLGA Nanoparticles

The PLGA nanoparticles were synthesized using an emulsion-solvent evaporation method^22,38^. Typically, 150 mg of PLGA (53/47, IV 1.03, PURAC, Gorinchem, Netherlands) was dissolved in 4 ml of dichloromethane (DCM, Sigma, St. Louis, Missouri, USA) at room temperature for 15 min. Subsequently, the organic phase was emulsified in 10 ml of an aqueous phase containing polyvinyl acetate (PVA, Sigma, St. Louis, Missouri, USA) as a stabilizer (2% w/v) using a Misonix sonicator (XL-2000, Millis, Massachusetts, USA) with remote operation approximately 30 times. The resulting o/w emulsion was stirred over night to facilitate the evaporation of the volatile DCM solvent. The PLGA nanoparticles were then collected after centrifugation at 11,000 rpm for 6 min. The loading of TA (Sigma, St. Louis, Missouri, USA) or coumarin 6 (Sigma, St. Louis, Missouri, USA) was achieved by dissolving the respective cargo with PLGA in DCM (20% for TA and 2% for coumarin 6, w/w of polymer), and the remaining procedure was performed as previously described. The cargo loaded PLGA nanoparticles were stored at 4 °C before use.

### Characterization of the Drug Loading Efficiency

To determine the loading efficiency of the TA-loaded microspheres, the TA was extracted and quantified by HPLC. For the TA extraction, 20 mg of the TA-loaded nanoparticles was dissolved in 1 ml of DMSO (Sigma, St. Louis, Missouri, USA). Subsequently, 9 ml of methanol (Sigma, St. Louis, Missouri, USA) was added to precipitate the polymer, and the suspension was shaken for 30 min. The residue was filtered, and the amount of TA was analyzed by HPLC using a Prominence autosampler equipped with a UV/VIS detector (Shimadzu-SPD M20A, Columbia, Maryland, USA). The drug quantification analyses were performed on a Poroshell 120 SB-C18 column (4.6 × 150 mm, particle diameter 2.7 μm) (Agilent Technologies, Santa Clara, California, USA). The mobile phase for the TA measurements was acetonitrile/H_2_O/trifluoroacetic acid (Sigma, St. Louis, Missouri, USA), 50:50:1 (v/v), at a flow rate of 0.5 ml/min. The run lasted 7 min, and the absorbance was measured at 240 nm. The loading efficiency of coumarin 6 was determined by fluorescence intensity as shown in in Supplementary information Table S2.

### Preparation of Structured Hydrogel via Microfluidics

Structured hydrogel particles were fabricated on a flow-focusing polydimethylsiloxane (PDMS) microchip, as illustrated in Fig. 1a and Figure S1 (supplementary information). The aqueous stream and oil stream were co-injected into the device using syringe pumps (PHD2000, Harvard Apparatus, Holliston, Massachusetts, UAS). The aqueous stream contained the PEGDA monomer (Mn = 575), alginate, EDTA-Ca and PLGA nanoparticles, and the aqueous stream was sheared by an oil stream (HFE-7500 with 1 wt% pico-surf) to form monodispersed pre-gel droplets. An aqueous flow rate of 5 μL/min and an oil flow rate of 30 μL/min generated 158 μm diameter droplets.

There was a two-step polymerization along with the droplet formation process. In the first step, the droplets were irradiated under UV-light (40–1151 mW/cm^2^, OmniCure S1500, Mississauga, Ontario, Canada,) to initiate the PEGDA polymerization. During the polymerization, the affinity between the poly(PEGDA) and PEGDA monomer changed, resulting in the phase separation between the poly(PEGDA) and PEGDA monomers in the aqueous solution. Accordingly, the poly(PEGDA) core formed in an alginate pre-gel droplet. Moreover, along with this process, the PLGA nanoparticles spontaneously aggregated in the poly(PEGDA) cores due to their similar wettability. In the second step, the droplets were collected in the HFE 7500 oil containing acetic acid (0.5 vol%) to initiate the alginate gelation by releasing Ca^2+^ from EDTA-Ca and form the core (poly(PEGDA))-shell (alginate) structured hydrogel particles with encapsulated PLGA nanoparticles in the core compartment. The remaining oil phase was washed off using a demulsifier to obtain the structured hydrogel particles. The hydrogel particles were stored at 4 °C before use.

### Characterization of Structured Hydrogel Particles

To visualize the PLGA nanoparticles in the PEGDA droplets, coumarin 6 was used to label the PLGA nanoparticles. The labeled particles had fluorescence signals (excitation wavelength 444 nm, emission wavelength 505 nm) that were measured via microscopy. The fluorescence signals were enhanced when the PLGA nanoparticles were trapped in the poly(PEGDA) core, and the signal intensity of the shell was measured and used to calculate the partition ratio. MATLAB was used to process the images (more details are provided in section 2 in the supplementary information). The data presented mean ± standard deviations.

For the coumarin 6 release test, the coumarin 6 labeled PLGA nanoparticles were placed into 1 ml PVA solution (0.5%, Mw 12K-23K) for well-dispersion of PLGA nanoparticles in a PVA solution. After that, 50 μL of PVA solution with PLGA nanoparticle suspension was mixed with 1 ml of a phosphate-buffered solution (PBS, pH 7.4) at 37 °C to evaluate its drug releasing profile. After pre-defined intervals, 20 μl of the solution collected was observed under microscopy to record the fluorescence images. After observation, the remaining sample was centrifuged, and the supernatant was removed and replaced with fresh PBS solution for next time point measurement. To determine the coumarin 6 concentration, the average fluorescence intensity was measured and calculated. To determine the release profile of TA, the prepared hydrogel particles were placed in 1 ml of PBS buffer (pH 7.4) at 37 °C. After pre-defined intervals, the samples were centrifuged, and the supernatant was collected and replaced with fresh PBS solution. The TA amount in the collected buffer was determined by HPLC, as previously described.

### *In vivo* evaluation

To evaluate the long-term therapeutic efficiency of using structured hydrogel particles, *in vivo* experiments were performed. The experimental protocol was approved by the Institutional Animal Care and Use Committee (IACUC) of the Singapore Health Services (SingHealth) and was in line with the SingHealth guidelines for the care and use of experimental animals. Six female New Zealand white rabbits (n = 6; 3 months of age, body weight of 4.0 ± 0.5 kg) were anesthetized and wounded. Six scars (length: 6 mm) were made on the ventral surface of each ear with the removal of the perichondrium. The epidermis, dermis, and perichondrium were thoroughly removed using a dissecting microscope. A liquid adhesive (LIQUIBAND, Advanced Medical Solutions (Plymouth) Limited, Devon, UK) was applied to the surrounding skin and was followed by wound coverage with a polyurethane dressing (Tegaderm, 3 M Health Care, St. Paul, Minnesota, USA). Then, the wound areas were examined every week to observe the curing process and scar formation. The polyurethane dressing remained in place at all times to ensure a moist wound environment. This dressing was removed when the wounds were fully epithelialized. The drug-loaded hydrogel particles were administered by subcutaneous injection to the rabbit ears, in which the wounds were created forming well-defined scarred areas. 100 μl of the solution with particle suspension (drug concentration 2.5 mg/mL) was then uploaded to the scarred area. Six treatment formulas were evaluated for comparison using this model. The formulas were normal saline, a TA suspension, the PLGA nanoparticles, the alginate hydrogel particles, the poly(PEGDA) hydrogel particles and the core/shell hydrogel particles. Normal saline was used the control experiment. The TA concentration in the other five formulas was 2.5 mg/ml.

The scars were harvested, bisected at their highest point, and processed for histological analysis using hematoxylin and eosin (H&E) stains 4 weeks’ post-injection. The scar elevation was quantified by measuring the SEI under 10× magnification. The SEI is the ratio of the tissue height in the total wound area and the tissue height of the normal tissue area beside the scar. The quantitative data in this study are presented as the mean ± standard deviation. The statistical analyses were performed using Origin Pro 2017. The t-test was used to analyze the parameters from different groups. The differences were considered significant when two-sided p < 0.05.

## Electronic supplementary material

Supporting information
