# Supplementary material for: Polymerization-Induced Phase Separation Formation of Structured Hydrogel Particles via Microfluidics for Scar Therapeutics
Source: Sci Rep. 2018 Feb 2;8:2245. doi: 10.1038/s41598-018-20516-9 (PMC5797090; doi:10.1038/s41598-018-20516-9)
Supplement: Supplementary file 1 — Supporting information [file 41598_2018_20516_MOESM1_ESM.pdf]

Supplementary Information

Polymerization-Induced Phase Separation Formation of Structured Hydrogel Particles via Microfluidics for Scar Therapeutics

S. Guo[1], G. Kang[2], D.-T. Phan[1], M. N. Hsu[1], Y. C. Por[2], & C. H. Chen[1,3,4]

[1]Department of Biomedical Engineering, National University of Singapore, 4 Engineering Drive 3, 117583 Singapore. [2]Department of Plastic, Reconstructive & Aesthetic Surgery, KK Women's and Children's Hospital, 100 Bukit Timah Rd, 229899, Singapore. [3]Biomedical Institute for Global Health Research and Technology (BIGHEART), National University of Singapore, 14 Medical Drive, 117599 Singapore. [4]Singapore Institute of Neurotechnology (SINAPSE), National University of Singapore, 28 Medical Drive, 117456 Singapore. Correspondence and requests for materials should be addressed to C. H. Chen (email: biecch@nus.edu.sg).

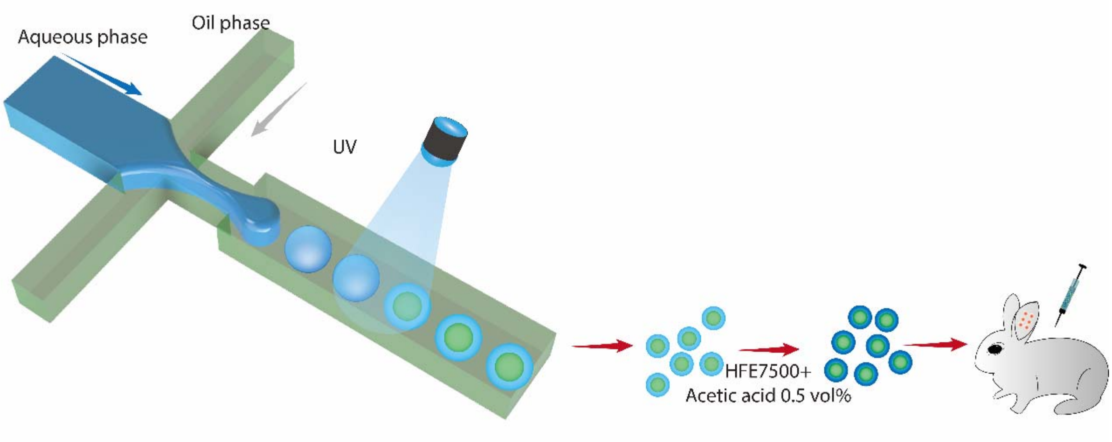

Figure S1 Polymerization induced phase separation to form core-shell structured hydrogel particle through microfluidics: Monodispersed pre-gel droplets containing PEGDA (Mn=575), Alginate, PLGA nanoparticles (loaded with drug), photo initiator and EDTA-Ca generated through flow focused microfluidic device were irradiated by UV light for polymerization forming core-shell hydrogel particles. To future gel aqueous droplets, HFE 7500 containing acetic acid 0.5 vol% was used to trigger subsequent crosslinking of alginate forming PEGDA core- alginate shell hydrogel particles for sustainable hydrophobic drug treatment *in vivo*.

Section 1 Conversion of PEGDA

The weight ratios of PEGDA cores in core-sell particles were measured to indicate the conversion of PEGDA. This conversion was calculated by gravimetric method, dividing the mass of PEGDA cores by the mass of total starting monomer. We firstly measured the volume of a given quality of the pre-gel aqueous solution to obtain its density. After polymerization, we collected the poly(PEGDA) cores. The mass of total starting monomer was estimated by 150 μl (5 μl/min×30 min) multiplied by the PEGDA concentration in the starting solution. Finally, the PEGDA cores were washed via ethanol by three times, and were weighted to obtain the mass of the polymerized PEGDA cores.

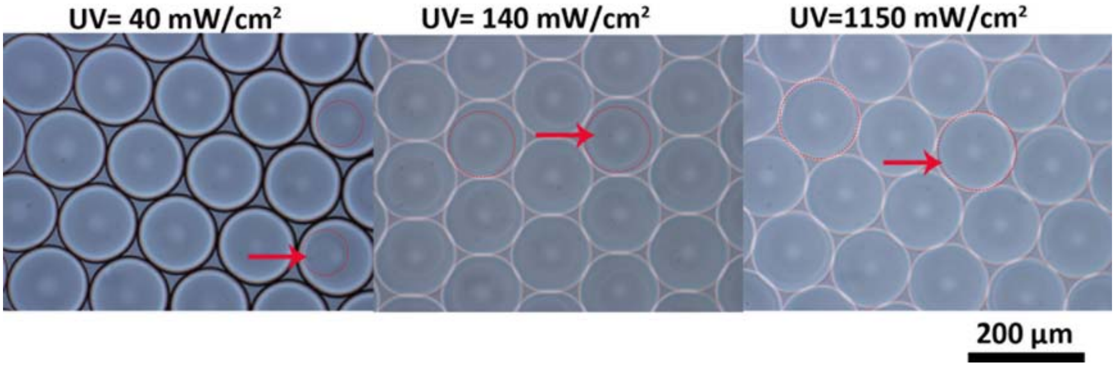

Figure S2 The high resolution images of core-shell particles with clear boundaries were recorded. In this experiment, PEGDA concentration was 5wt%.

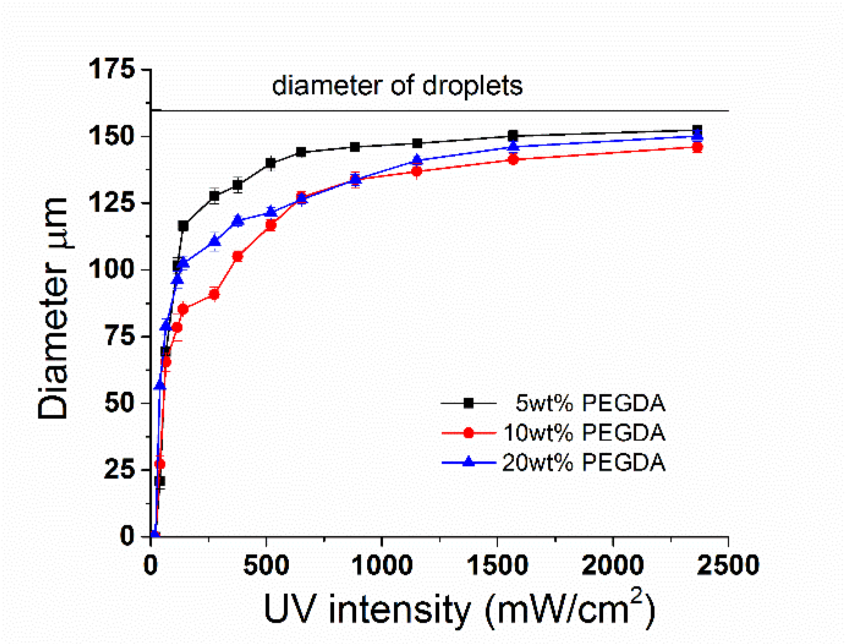

Figure S3 The diameters of poly(PEGDA) cores during UV-polymerizations were measured in the systems with different PEGDA monomer concentrations (5 wt% - black squares, 10 wt% - red dot and 20 wt% - blue triangle). Error bars here was standard deviation of the poly(PEGDA) core mean diameters.

Table S1 The conversion of PEGDA (%)

	UV output intensity mW/cm^2					
PEGDA content	40	140	230	367	520	1151
5 wt%	12	43	71	76	84	98
10 wt%	11	38	62	74	86	98
20wt%	11	39	65	75	85	99

Section 2 The mechanism of polymerization induced phase separation of PEGDA

During UV induced polymerization, the free radicals (R•) were generated via UV decomposition of photo initiators. R• was capable of cleaving C=C bond to give a free-radical initiation site, forming monomer radical (Figure S4). These monomer radicals then reacted with PEGDA monomer forming oligomers, a polymeric network. Therefore, in our experiment, within water-in-oil droplets, oligomers aggregated together to be cross-linked with each other, forming poly(PEGDA) beads. The increasing molecular weight of poly(PEGDA) decreased the compatibility between poly(PEGDA) and water causing phase separation. Thus water was repelled from the poly(PEGDA) forming the droplets with core-shell structure [1]. Based on this mechanism, increasing UV intensity resulted in increasing R• concentration. Both polymer numbers and polymer weight would increase (the concentrations of monomer radicals/oligomers increase) forming a polymeric network. Therefore, poly(PEGDA) particles were formed in the aqueous droplet, which was consist with the previous work [1].

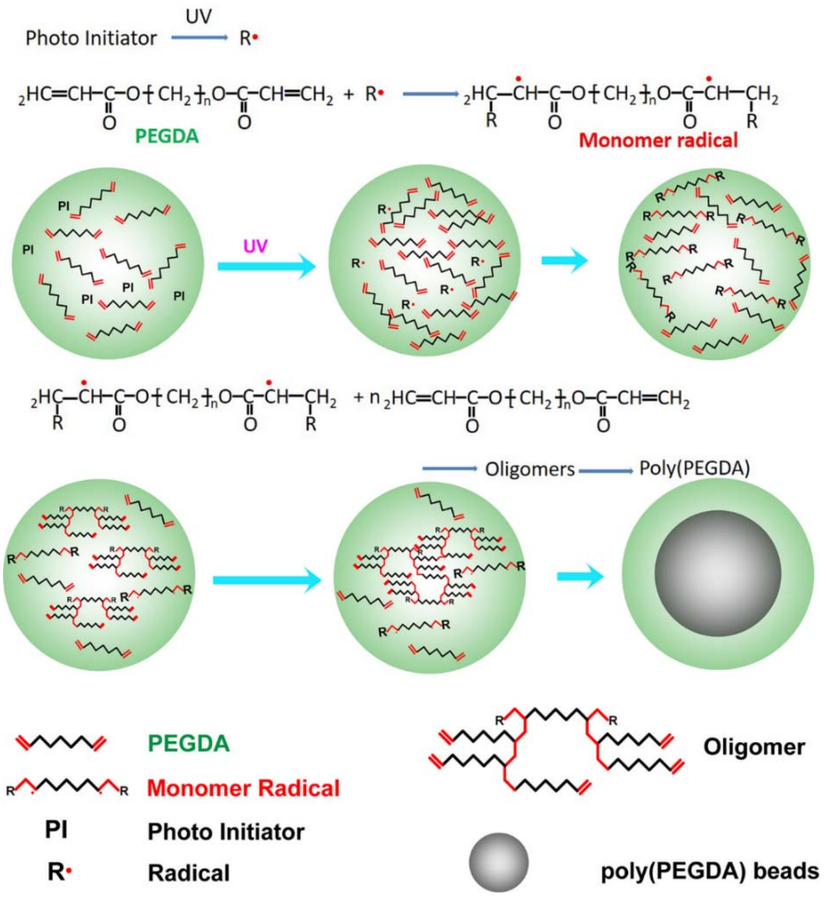

Figure S4 Scheme of the photo-initiated polymerization of monomer PEGDA.

Section 3 The partition ratio of PLGA-- MATLAB procedure for image processing

1. Droplet diameter

The surface area of the formed droplet was evaluated using a customized MATLAB routine. The program converted the recorded greyscale images into binary images basing on a certain threshold value and extracted the biggest area of the region of interest, which consists of a formed droplet. The equivalent radius was determined as the radius of a circle with the same surface area of the biggest area.

Radius = sqrt(biggestArea/pi)/ratio;

The calibration ratio is used to convert the radius back to the length unit (in micrometer).

2. Core-shell fluorescence intensity

To evaluate the relative change of the fluorescence intensity over time, we kept a fix threshold value (i.e. reference value) when converting the fluorescence images to binary images. Since the recorded images show different fluorescence intensities over time, the fluorescence intensity is only evaluated properly by using the same reference value. In details, this threshold value is optimized to minimum the noise of the whole set of the binary images. Then, the background (i.e. outer regions) will be black and the droplet/core area becomes totally white. Next, we extract the biggest area of the region of interest (i.e. white area), which consists of a droplet/core. Also, we can convert this area from the number of pixels into millimeter square using a calibration ratio, which is determined based on the given magnification of the imaging system.

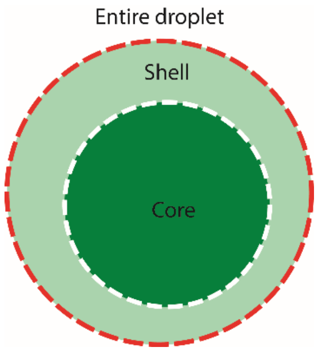

Figure S5 Illustration of the detected boundaries of core/shell model.

$$\overline{intensity}_{shell} = \frac{\overline{intensity}_{drop} \times area_{drop} - \overline{intensity}_{core} \times area_{core}}{area_{drop} - area_{core}}$$

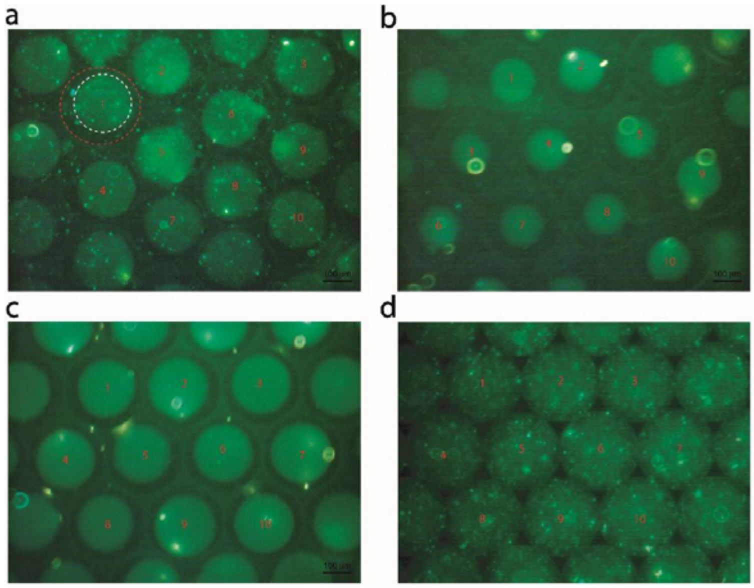

Figure S6 An example of images was used for intensity measurement. The droplets were numbered correspondingly. For each image, ten droplets were extracted to get the average value and its standard deviation.

To estimate the shell area/fluorescence intensity, we first extract the core and overall droplet boundary using an aforementioned adaptive threshold value. Next, the fluorescence intensity of core and entire droplet were measured based on the detected boundaries (Figure S5). The average fluorescence intensity of shell is calculated by the following equation.

Table S2 Details of courmarin 6 and TA loading

	Loading Efficiency	Loading Amount (mg/mgPLGA)	Concentration in hydrogel (mg/ml)
Courmarin 6	4.25%	7.08×10^{-3}	8.8×10^{-4}
TA	78.92%	0.105	2.5

Section 4 In vitro drug release test

The release rate (after burst release) were calculated by Oringin Pro 2017. The results were showed in Figure S7. The release from core-shell hydrogel particles each day was between 1-3% for 3 weeks, while the release from other formulas decreased significantly to 0% in the first 5 days. In this plot, the advantage of presented core-shell particle for long term stable hydrophobic reagent releasing was highlighted.

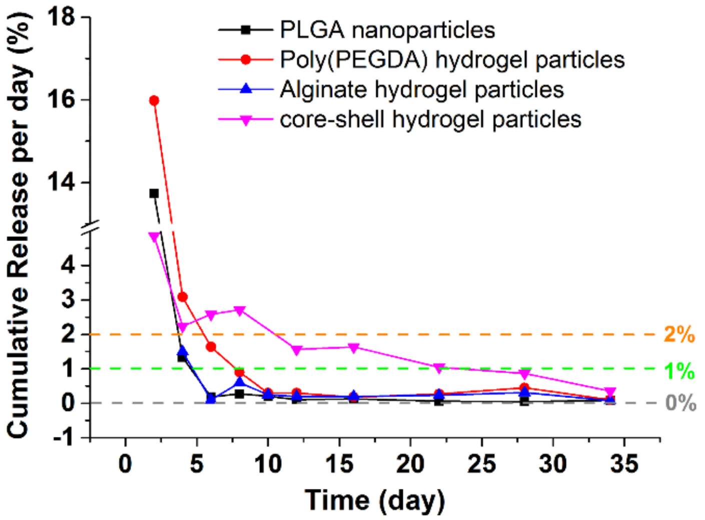

Figure S7 Cumulative release rates at every detection interval (After burst release) from PLGA nanoparticles, alginate hydrogel particles, poly(PEGDA) hydrogel particles and core-shell hydrogels were observed to indicate the advantage of stable long term hydrophobic reagent releasing by using core-shell structured hydrogel particles.

Table S3 SEI analysis results

Formulas	SEI<br>(means ± standard deviation)	*p* value
Normal Saline	1.81±0.18	-
TA	1.71±0.14	0.02
PLGA	1.68±0.13	0.03
Alginate hydrogel	1.63±0.12	0.01
Poly(PEGDA) hydrogel	1.54±0.15	0.02
Core-shell hydrogel	1.39±0.11	0.01

Section 5 Effect of alginate and poly(PEGDA) on the scar treatment

The foreign body reactions of blank alginate and blank poly(PEGDA) were estimated. The SEI values of the scars treated by blank alginate and poly(PEGDA) were similar with the value of the one treated by normal saline. The results of these control experiments suggested that the foreign body reactions of alginate and poly(PEGDA) were not significant to effect scar treatment using presented core-shell hydrogel particles.

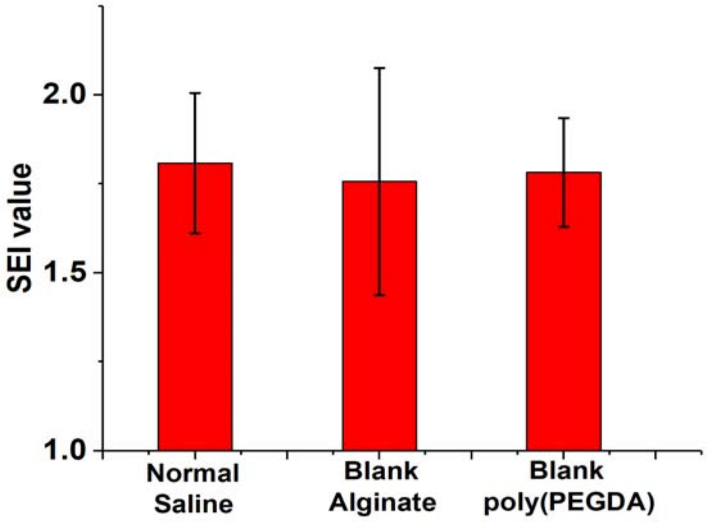

Figure S8 SEI data for the wounds treated with normal saline, blank alginate and blank poly(PEGDA) without drug. * $p < 0.05$, $n = 12$. The error bars here are the standard deviations.

The additional histological slices with particles were shown in Figure S9a. The particle was identified by a round empty circle surrounded by the dark dots. By comparing with the normal fat cells (Figure S9b), which exhibited irregular shape and close-packed structures, the remained hydrogel particle was identified by the empty circle (pointed out by a red arrow) in Figure S9a. The surrounded dark dots around the empty circle suggested the effect of slight foreign body reaction.

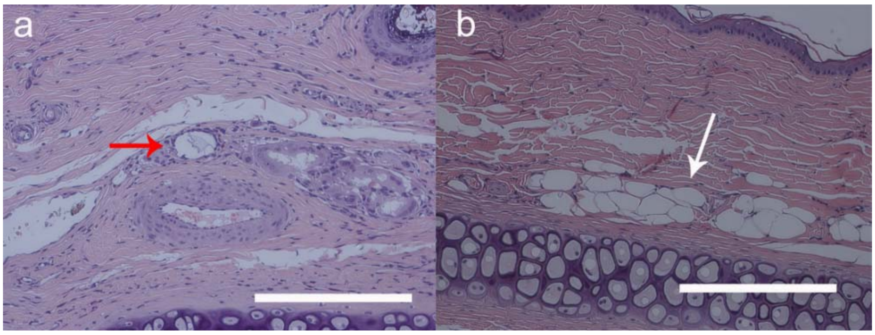

Figure S9 Images of hydrogel (a) and fat cells (b) from the scars 4 weeks after the injection of the drug-loaded hydrogels. The bar inserted is 500 μm.

Reference:

[1]. Guo, S., Yao, T., Ji, X.B., Zeng, C.F., Wang, C. Q. & Zhang, L. X. Versatile preparation of nonspherical multiple hydrogel core pam/peg emulsions and hierarchical hydrogel microarchitectures. *Angew. Chem. Int. Ed.* 2014 53, 7504-7509.
